# Cancer Stigma, Distress, and Cancer Coping in Relation to the Quality of Life Among Patients with Lung Cancer: A Cross-Sectional Study

**DOI:** 10.3390/healthcare14193163

**Published:** 2026-09-24

**Authors:** Ji-Yeon Pyo, Seungkyoung Yang

**Affiliations:** 1 Department of Nursing, Pusan National University Yangsan Hospital, Yangsan 50612, Republic of Korea; 2 Department of Nursing, Kyungnam University, Changwon 51767, Republic of Korea

**Keywords:** lung cancer, cancer stigma, distress, cancer coping, quality of life

## Abstract

**Background/Objectives**: Patients with lung cancer often experience impaired quality of life because of symptom burden and psychosocial difficulties. Cancer stigma and distress are associated with poorer quality of life, whereas adaptive coping may support psychological adjustment. This study examined the relationships among cancer stigma, distress, cancer coping, and quality of life and identified factors associated with quality of life in patients with lung cancer. **Methods**: This cross-sectional correlational study included 168 patients with lung cancer receiving inpatient or outpatient care at a university hospital in the Republic of Korea. Data were collected from 1 August to 1 September 2025, using structured self-report questionnaires. Descriptive statistics, group comparisons, Pearson correlations, and hierarchical multiple regression were performed. **Results**: The mean scores for cancer stigma, distress, cancer coping, and quality of life were 46.43 ± 8.46, 4.49 ± 2.53, 56.43 ± 15.07, and 74.02 ± 14.93, respectively; 57.1% of participants reported clinically significant distress. Quality of life was negatively correlated with cancer stigma (r = −0.63, *p* < 0.001) and distress (r = −0.76, *p* < 0.001) and positively correlated with cancer coping (r = 0.71, *p* < 0.001). Cancer coping, current activity level, and distress were independently associated with quality of life. The final model explained 75.2% of the variance, with cancer coping showing the largest standardized coefficient. **Conclusions**: Better cancer coping, higher activity levels, and lower distress were associated with better quality of life. Psychosocial nursing care should support adaptive coping, functional activity, and distress management.

## 1. Introduction

According to the Korean national cancer statistics for 2023, lung cancer ranked second in incidence both overall and among men and fourth among women [1]. The 5-year relative survival rate for lung cancer increased from 27.7% in 2011–2015 to 42.5% in 2019–2023 [1]. This improvement in survival indicates that patients are living longer after a lung cancer diagnosis, highlighting the growing importance of symptom management and quality of life enhancement during and after cancer treatment.

Patients with lung cancer have been reported to experience poorer quality of life than those with other types of cancer [2]. Among patients with advanced lung cancer, quality of life may deteriorate across successive lines of palliative chemotherapy and may decline further as the disease progresses toward the end of life [3,4]. In particular, respiratory symptoms such as dyspnea and cough impose a substantial symptom burden, and inadequately controlled symptoms can compromise quality of life [5,6,7]. In addition, psychosocial problems, including anxiety, depression, social isolation, and financial difficulties, can restrict social roles and increase psychological burden and stress, thereby reducing quality of life [8,9,10]. Therefore, improving quality of life among patients with lung cancer requires attention to both symptom burden and psychosocial factors. Among these psychosocial factors, cancer stigma is particularly relevant in lung cancer because of the persistent social attribution of the disease to cigarette smoking and its potential consequences for psychological well-being and quality of life.

Cancer stigma refers to the experience of rejection, blame, or devaluation related to a cancer diagnosis [11]. Because lung cancer is commonly associated with cigarette smoking, patients may experience internalized stigma, including shame, guilt, regret, and self-blame, as well as externalized stigma, including perceived judgment, blame, and negative evaluation by others. A recent systematic review of 34 studies found that lung cancer stigma is associated with a broad range of demographic, clinical, psychological, behavioral, and social factors, underscoring its importance for patient well-being [12]. Patients with lung cancer have also been reported to experience greater stigma than patients with other types of cancer [13,14]. Moreover, perceived stigma is associated with poorer quality of life among patients with lung cancer [15,16]. Importantly, stigma may also be linked to other psychosocial processes relevant to quality of life. Experiences of blame, shame, social isolation, and perceived judgment may contribute to emotional distress, which may in turn influence how patients cope with cancer and its treatment.

Psychological distress is defined as an unpleasant emotional experience that may interfere with a patient’s ability to cope effectively with cancer and its treatment [17]. Distress is an important concern in cancer care because severe or persistent distress may adversely affect psychological adjustment, treatment-related decision making, and overall well-being [17,18,19,20]. Accordingly, early identification and management of distress are important for maintaining psychological adjustment and quality of life among patients with lung cancer.

Cancer coping refers to the process by which individuals use available resources to alleviate cancer-related stress and achieve physical and psychological adaptation [21]. Cancer coping can be divided into intrapersonal and interpersonal coping. Intrapersonal coping refers to the ways in which patients independently respond to crises and primarily includes problem-focused strategies such as positive reframing, active coping, and planning [21]. Interpersonal coping involves obtaining emotional comfort and seeking solutions through interactions with family members, healthcare professionals, and support groups. Social support can facilitate both emotion-focused and problem-focused coping [21]. The use of adaptive coping strategies is associated with better psychological adjustment and quality of life among patients with cancer [22,23]. Emotion-focused coping may provide a temporary buffering effect during the early phase of cancer diagnosis; however, problem-focused coping and the use of social support have been reported to be more effective throughout the longer-term treatment process [24]. These findings suggest that cancer coping extends beyond a simple psychological response and may represent an important modifiable factor associated with the treatment process and quality of life.

Previous studies have shown that higher perceived stigma among patients with lung cancer is associated with greater depression and anxiety and poorer quality of life [14,15,16]. Psychological distress may interfere with patients’ ability to cope effectively with cancer and its treatment [25]. Cancer coping is associated with psychosocial adjustment and quality of life among patients with cancer [22,23,26]. Taken together, cancer stigma, psychological distress, and cancer coping represent interrelated psychosocial factors that may be associated with quality of life among patients with lung cancer. However, evidence examining these factors simultaneously in relation to quality of life in this population remains limited. Therefore, this study aimed to assess the levels of cancer stigma, psychological distress, cancer coping, and quality of life and to examine the relationships among these variables. We hypothesized that cancer stigma and psychological distress would be negatively associated with quality of life, whereas cancer coping would be positively associated with quality of life and would contribute additional explanatory value after sociodemographic and clinical factors, cancer stigma, and psychological distress were considered.

## 2. Materials and Methods

### 2.1. Design and Setting

This cross-sectional correlational study examined the associations of cancer stigma, psychological distress, and cancer coping with quality of life among patients with lung cancer. Data were collected from 1 August to 1 September 2025, from patients receiving inpatient treatment in the lung cancer ward or outpatient care at the respiratory center of Pusan National University Yangsan Hospital, Yangsan, Republic of Korea. This study was reported in accordance with the Strengthening the Reporting of Observational Studies in Epidemiology (STROBE) guidelines. 

### 2.2. Sample Size

The required sample size was estimated using G*Power version 3.1.9.7. To ensure adequate power for the planned multivariable analysis, the calculation was conducted conservatively for up to 19 predictors, with a significance level of 0.05, a medium effect size (f^2^) of 0.15, and statistical power of 0.80. The analysis indicated that a minimum of 153 participants was required. To allow for incomplete questionnaires, 168 consenting participants received the questionnaire. All 168 questionnaires were returned complete, and no cases were excluded because of missing or incomplete data; therefore, 168 participants were included in the final analysis.

### 2.3. Inclusion Criteria

Participants were adults aged 18 years or older who had been diagnosed with lung cancer, were aware of their diagnosis, were able to understand the study information and questionnaire items, and provided written informed consent.

### 2.4. Exclusion Criteria

Participants were excluded when severe cognitive, psychological, or clinical conditions prevented them from understanding or completing the questionnaire.

### 2.5. Measurements

#### 2.5.1. General and Disease-Related Characteristics

General and disease-related characteristics were assessed using a structured questionnaire. These included gender, age, education level, marital status, religion, employment status, monthly household income, perceived health status, time since lung cancer diagnosis, type and stage of lung cancer, comorbidities, treatment history, smoking status, current activity level, and family history of lung cancer. Current activity level was assessed using a self-reported item based on the Eastern Cooperative Oncology Group Performance Status (ECOG PS), originally described by Oken et al. [27] and previously used in patients with lung cancer [28]. The item comprised five categories corresponding to ECOG PS grades 0–4: (0) as active as before the illness, (1) mild symptoms but fully ambulatory, (2) ambulatory for more than half of the day, (3) confined to bed for more than half of the day, and (4) confined to bed throughout the day. Higher grades indicated poorer functional status.

#### 2.5.2. Cancer Stigma

Cancer stigma was measured using the Cataldo Lung Cancer Stigma Scale (CLCSS), developed by Cataldo et al. [29]. The instrument uses a 4-point Likert scale, with higher scores indicating greater levels of cancer stigma. In the original development study, construct validity was supported by exploratory factor analysis and by associations with theoretically related constructs, including self-esteem, depression, social support, and social conflict [29]. The Korean version used by Lee and Kim [14] was developed through translation and back-translation, pilot testing with patients with lung cancer, and expert review to establish content validity. Cronbach’s α was 0.96 in the original study [29], 0.85 in the Korean study [14], and 0.86 in the present study.

#### 2.5.3. Distress

Distress was assessed using the Korean-language version of the Distress Thermometer and Problem List, Version 2, provided by the National Comprehensive Cancer Network (NCCN) for distress screening among patients with cancer [30]. The instrument consists of the Distress Thermometer and a Problem List.

The Distress Thermometer is a visual analog scale on which participants indicate the level of distress experienced during the previous week, ranging from 0 (“no distress”) to 10 (“extreme distress”), with higher scores indicating greater psychological distress. The NCCN recommends a cutoff score of 4. Participants with scores below 4 were classified as being below the cutoff for clinically significant distress, whereas those with scores of 4 or higher were classified as having clinically significant distress.

The Problem List consists of 39 items across five domains: practical problems (6 items), family problems (4 items), emotional problems (6 items), spiritual or religious concerns (1 item), and physical problems (22 items). For each item, participants indicated whether they had experienced the problem during the previous week by responding “yes” or “no”. The Kuder–Richardson 20 coefficient for the 39-item Problem List was 0.91 in the present study.

#### 2.5.4. Cancer Coping

Cancer coping was measured using the Korean version of the Cancer Coping Questionnaire (K-CCQ), which was adapted and modified by Kim et al. [31] based on the Cancer Coping Questionnaire (CCQ) developed by Moorey et al. [21]. The K-CCQ consists of 23 items comprising two major domains: intrapersonal coping (14 items) and interpersonal coping (9 items). Intrapersonal coping includes positive reframing, active coping, and planning. Each item is rated on a 4-point Likert scale, with higher scores indicating greater use of adaptive coping strategies. Construct validity of the Korean version was supported by factor analysis and by significant correlations with established measures of active cognitive and behavioral coping strategies [31]. In the Korean validation study, Cronbach’s α was 0.90 for the total scale, 0.88 for intrapersonal coping, and 0.90 for interpersonal coping [31]. In the present study, Cronbach’s α was 0.95 for the total scale, 0.93 for intrapersonal coping, and 0.92 for interpersonal coping.

#### 2.5.5. Quality of Life

Quality of life was measured using the Korean version of the Functional Assessment of Cancer Therapy–Lung, Version 4 (FACT-L), originally developed by Cella et al. [32] and translated and validated in Korean by Yoo et al. [33]. The instrument assesses physical, social/family, emotional, and functional well-being, as well as lung cancer-specific symptoms and concerns. Items are rated on a 5-point Likert scale, with higher scores indicating better quality of life. The Korean version was translated and cross-culturally adapted using the Functional Assessment of Chronic Illness Therapy translation methodology. Its validity was supported by good convergent and divergent validity in comparisons with the Functional Living Index–Cancer and the Profile of Mood States [33]. In the Korean validation study, Cronbach’s α coefficients for the FACT-L scales ranged from 0.52 to 0.84 [33]. Cronbach’s α for the total scale was 0.75 in the present study.

### 2.6. Data Collection

Data were collected from patients with lung cancer receiving inpatient or outpatient care at Pusan National University Yangsan Hospital. Participants were recruited through convenience sampling based on voluntary participation. After obtaining cooperation from the nursing department and relevant clinical departments, recruitment notices were posted at the entrances of the inpatient wards and outpatient clinic. Patients who expressed interest in participating were screened by the researcher for eligibility according to the predefined inclusion and exclusion criteria. Eligible patients who agreed to participate provided written informed consent and completed a self-administered questionnaire, which took approximately 15 min. Assistance was provided as needed to participants who had difficulty reading or completing the questionnaire independently. Completed questionnaires were placed in sealed envelopes and collected immediately to maintain confidentiality, and participants received a small token of appreciation. As recruitment relied on voluntary responses to posted notices and patients were not individually approached, the total number of potentially eligible patients who were exposed to the recruitment notices, as well as the numbers who declined participation or did not respond, were not systematically recorded.

### 2.7. Ethical Considerations

The study was conducted in accordance with the Declaration of Helsinki and was approved by the Institutional Review Board of Pusan National University Yangsan Hospital (IRB No. 11-2025-035; approved on 24 June 2025). Cooperation was also obtained from the nursing department and relevant clinical departments. Before data collection, participants were informed of the study purpose and procedures, the voluntary nature of participation, confidentiality and anonymity, and their right to withdraw at any time without disadvantage. Written informed consent was obtained from all participants.

All collected data were stored securely in locked cabinets and password-protected electronic files accessible only to the researcher. In accordance with the Personal Information Protection Act, the data will be retained for 3 years, after which paper records will be shredded and electronic files permanently deleted to prevent recovery.

### 2.8. Statistical Analysis

The collected data were analyzed using SPSS/WIN version 25.0. Descriptive statistics were calculated to summarize participants’ general and disease-related characteristics and the distributions of the major study variables. Differences in quality of life according to general and disease-related characteristics were analyzed using independent *t*-tests and one-way analysis of variance, followed by Scheffé’s post hoc tests. Pearson’s correlation coefficients were calculated to examine the relationships among the major study variables. Hierarchical multiple regression analysis was performed to evaluate the incremental associations of psychosocial variables with quality of life. Model 1 included spouse status, perceived health status, type of lung cancer, cancer stage, comorbidities, chemotherapy, and current activity level as sociodemographic and clinical covariates selected based on a combination of clinical relevance, previous evidence, and univariable associations with quality of life. Cancer stigma was added in Model 2, distress in Model 3, and cancer coping in Model 4 to assess whether coping contributed additional explanatory value beyond the variables entered in the preceding models. Categorical predictors were entered as dummy variables. Multicollinearity was assessed using tolerance and variance inflation factor values, and the independence of residuals was assessed using the Durbin–Watson statistic. Statistical significance was set at *p* < 0.05.

## 3. Results

### 3.1. General and Disease-Related Characteristics of the Participants

Among the 168 participants, 72.0% (*n* = 121) were men and 28.0% (*n* = 47) were women. The mean age was 66.71 ± 8.94 years, and participants aged 70 years or older constituted the largest age group (42.3%, *n* = 71). Regarding educational attainment, high school graduates comprised the largest group (40.5%, *n* = 68). Most participants had a spouse (79.2%, *n* = 133), had no religious affiliation (60.7%, *n* = 102), and were unemployed (71.4%, *n* = 120). The mean monthly household income, including family income, was KRW 2.72 ± 1.38 million, and the largest proportion reported a monthly income of KRW 2.0 to less than 4.0 million (72.0%, *n* = 121). Poor perceived health status was the most frequently reported category (35.2%, *n* = 59).

Regarding disease-related characteristics, 47.0% (*n* = 79) had been diagnosed with lung cancer less than 12 months previously. Most participants had non-small cell lung cancer (76.8%, *n* = 129), and stage IV disease was the most common cancer stage (30.3%, *n* = 51). Regarding treatment history, 51.2% (*n* = 86) had not undergone surgery, 81.5% (*n* = 137) had received chemotherapy, and 79.2% (*n* = 133) had not received radiation therapy. Former smokers constituted the largest smoking-status group (61.9%, *n* = 104). Regarding current activity level, participants who had mild symptoms but remained fully ambulatory comprised the largest group (46.4%, *n* = 78). Most participants had no family history of lung cancer (89.3%, *n* = 150) (Table 1).

### 3.2. Levels of Cancer Stigma, Distress, Cancer Coping, and Quality of Life

The mean cancer stigma score was 46.43 ± 8.46. Among the subdomains, the mean scores were 10.21 ± 2.94 for smoking-related stigma, 17.02 ± 3.98 for social isolation, 6.43 ± 1.35 for discrimination, and 12.77 ± 2.81 for stigma and shame.

The mean Distress Thermometer score was 4.49 ± 2.53. Using a cutoff score of 4, 42.9% (*n* = 72) of participants scored below the cutoff, whereas 57.1% (*n* = 96) scored 4 or higher, indicating clinically significant distress. The mean number of problems reported on the Problem List was 11.38 ± 7.62. The mean numbers of problems by domain were 2.61 ± 2.04 for emotional problems, 6.98 ± 4.57 for physical problems, 1.24 ± 1.37 for practical problems, 0.53 ± 0.91 for family problems, and 0.01 ± 0.07 for spiritual or religious concerns.

The mean cancer coping score was 56.43 ± 15.07. The mean subdomain scores were 22.68 ± 7.69 for interpersonal coping and 33.76 ± 8.62 for intrapersonal coping.

The mean quality-of-life score was 74.02 ± 14.93. The mean subdomain scores were 17.47 ± 7.39 for physical well-being, 14.39 ± 5.83 for emotional well-being, 16.14 ± 5.69 for social/family well-being, 13.27 ± 1.73 for functional well-being, and 12.74 ± 2.75 for the lung cancer subscale (Table 2).

### 3.3. Differences in Quality of Life According to Participants’ General and Disease-Related Characteristics

Quality of life differed significantly according to spouse status (t = 2.10, *p* = 0.037), perceived health status (F = 78.40, *p* < 0.001), type of lung cancer (t = 2.39, *p* = 0.029), cancer stage (F = 5.68, *p* = 0.001), comorbidities (t = −2.10, *p* = 0.037), chemotherapy (t = −3.74, *p* < 0.001), and current activity level (F = 30.63, *p* < 0.001). Scheffé’s post hoc test showed that participants with stage I cancer had significantly higher quality-of-life scores than those with stage IV cancer. Participants who were as active as before the illness or had only mild symptoms had significantly higher quality of life than those with activity limitations (Table 3).

### 3.4. Correlations Among Cancer Stigma, Distress, Cancer Coping, and Quality of Life

Table 4 presents the correlations among cancer stigma, distress, cancer coping, and quality of life. Quality of life was significantly negatively correlated with psychological distress (r = −0.76, *p* < 0.001) and cancer stigma (r = −0.63, *p* < 0.001), whereas it was significantly positively correlated with cancer coping (r = 0.71, *p* < 0.001).

### 3.5. Factors Associated with Quality of Life

Hierarchical multiple regression analysis was conducted to examine whether cancer stigma, distress, and cancer coping were independently associated with quality of life after adjustment for demographic and clinical covariates. Tolerance values ranged from 0.26 to 0.96 and variance inflation factor values ranged from 1.047 to 3.832, indicating no evidence of problematic multicollinearity. The Durbin–Watson statistic was 1.919, which was close to 2 and supported independence of the residuals.

In Model 1, which included sociodemographic and disease-related characteristics, current activity level was significantly associated with quality of life. The model explained 57.9% of the variance in quality of life and was statistically significant (F = 16.32, *p* < 0.001).

In Model 2, cancer stigma was added, resulting in a 6.4 percentage-point increase in explained variance and a total explained variance of 64.3% (F = 19.70, *p* < 0.001). Higher cancer stigma and lower current activity levels were significantly associated with poorer quality of life.

In Model 3, the addition of distress increased the explained variance by 4.3 percentage points, yielding a total explained variance of 68.6% (F = 22.16, *p* < 0.001). Higher distress, greater cancer stigma, and lower current activity levels were significantly associated with poorer quality of life.

In Model 4, cancer coping was added, increasing the explained variance by 6.6 percentage points to 75.2% (F = 28.61, *p* < 0.001). Greater cancer coping was associated with better quality of life (β = 0.37, *p* < 0.001), whereas higher distress was associated with poorer quality of life (β = −0.27, *p* < 0.001). Compared with participants who were as active as before the illness, all four categories indicating lower activity were associated with poorer quality of life (β = −0.28 to −0.13, p = 0.001–0.043). Cancer stigma was no longer statistically significant in the final model (β = −0.06, *p* = 0.317). Cancer coping showed the largest standardized regression coefficient in the final model. The detailed regression results are presented in Table 5.

## 4. Discussion

The mean cancer stigma score among patients with lung cancer in the present study was 46.43 ± 8.46. According to a recent systematic review, patients with lung cancer experience not only internalized stigma, such as smoking-related self-blame, shame, and guilt, but also externalized stigma, including blame, negative judgment, and discrimination by others. Lung cancer stigma has also been associated with greater psychological distress, poorer emotional functioning, and lower quality of life [12].

Because lung cancer is commonly perceived as being closely associated with smoking, patients may attribute responsibility for the development of the disease to themselves or experience blame and negative judgment from others. Smoking-related stigma was also identified in the present study. However, recent evidence suggests that lung cancer stigma is not limited to current or former smokers and may be associated with poorer emotional functioning regardless of smoking history [34]. Therefore, to reduce cancer stigma among patients with lung cancer, smoking-related self-blame, shame, perceived discrimination, and social isolation should be assessed early, and nonjudgmental communication and emotional support should be provided. Multidimensional nursing interventions that strengthen family and social support systems are also needed.

The mean Distress Thermometer score in the present study was 4.49 ± 2.53, and 57.1% of participants experienced clinically significant distress. A meta-analysis of studies that used the Distress Thermometer among patients with lung cancer reported a pooled prevalence of clinically significant distress of 49.04%, which was comparable to the finding of the present study [35]. In addition, a recent study of 435 patients with lung cancer found that 52.87% experienced psychological distress and identified symptom burden, avoidant coping styles, and socioeconomic factors as important correlates of distress [36]. Another study using the Distress Thermometer and Problem List suggested that physical and emotional problems identified during distress screening may help identify patients at risk of poorer prognosis [37]. These findings highlight the need for routine distress screening among patients with lung cancer and for the early identification of the physical, emotional, practical, and social problems underlying distress so that appropriate interventions can be provided.

The mean cancer coping score was 56.43 ± 15.07, which was slightly lower than that reported in a previous study of patients with lung cancer [38]. In the present study, 56.5% of the participants had stage III or higher disease, whereas only 11% had stage III or higher disease in the study by Oh [38], indicating a difference in the distribution of cancer stage.

Differences in age and disease-related characteristics may also have contributed to variation in coping, as age and clinical characteristics have been associated with coping strategies among patients with cancer [39,40]. Intrapersonal coping involves internal psychological processes such as thoughts, emotions, and attitudes, whereas interpersonal coping involves coping through relationships and interactions with others [21]. Accordingly, interventions should support both positive cognitive and emotional adjustment and effective use of family, professional, and social support.

The mean quality of life score in the present study was 74.02 ± 14.93. Previous studies reported scores of 79.90 ± 15.84 among patients receiving immunotherapy [41] and 66.43 ± 23.67 among those undergoing chemotherapy [42]. These differences may reflect variations in age, employment status, treatment modality, and cancer stage. As lung cancer progresses, increasing symptom burden, including dyspnea, fatigue, pain, and appetite loss, may impair overall quality of life and physical, emotional, role, and social functioning [5,6,43]. Performance status, comorbidities, treatment-related adverse effects, physical activity, and body weight have also been associated with quality of life [44]. Therefore, individualized interventions should consider cancer stage, functional status, symptom burden, and treatment modality.

The lung cancer subscale score was 12.74 ± 2.75. Because this subscale reflects lung cancer-specific symptoms and concerns, the finding reinforces the importance of assessing disease-specific symptom burden alongside general physical, emotional, social/family, and functional well-being. Comprehensive supportive care should integrate symptom assessment, rehabilitation, nutritional support, psychological screening, stigma-sensitive communication, social support, and adaptive coping throughout treatment and survivorship [34,45,46].

Quality of life differed significantly according to spouse status, perceived health status, type of lung cancer, cancer stage, comorbidities, chemotherapy, and current activity level. Participants with a spouse reported better quality of life, suggesting that family support may be relevant to psychosocial adjustment. In addition, perceived health status appeared to be an important factor related to quality of life. However, these univariable findings should be interpreted cautiously because several sociodemographic and disease-related characteristics were no longer statistically significant after adjustment in the hierarchical regression model.

Quality of life was also higher in the univariable analyses among participants with non-small cell lung cancer, earlier-stage disease, no comorbidities, and no history of chemotherapy. Previous studies have similarly linked symptom burden, advanced disease, comorbidity, and treatment-related adverse effects with poorer quality of life [47]. These findings support individualized assessment of clinical status and treatment burden rather than interpretation of any single clinical characteristic in isolation.

Current activity level showed a particularly consistent association with quality of life. Participants who remained as active as before the illness or had only mild symptoms reported better quality of life in the group comparisons, and lower activity remained independently associated with poorer quality of life in the final regression model. Regular assessment of functional activity and tailored rehabilitation or exercise support may therefore be useful components of supportive care.

Quality of life was significantly negatively correlated with distress and cancer stigma and positively correlated with cancer coping. These findings are consistent with previous studies reporting that higher levels of distress and cancer stigma are associated with poorer quality of life among patients with lung cancer [48]. Patients with lung cancer may experience substantial distress because of physical symptoms and uncertainty regarding prognosis, while smoking-related self-blame and social prejudice may also be associated with poorer quality of life.

In contrast, cancer coping was positively correlated with quality of life, consistent with findings among patients with advanced lung cancer [49]. Adaptive coping strategies, particularly positive reframing, may be associated with better quality of life among patients with advanced lung cancer, with anxiety and depressive symptoms mediating this relationship [49]. Therefore, integrated psychosocial interventions are needed to assess psychological distress and cancer stigma at an early stage, reduce self-blame and emotional distress, and strengthen adaptive coping strategies.

Cancer coping, current activity level, and distress were independently associated with quality of life, and the final regression model explained 75.2% of the variance. Cancer coping showed the largest standardized coefficient. This finding is consistent with recent studies showing that positive coping is associated with better quality of life, whereas negative or acceptance–resignation coping is associated with poorer quality of life and may mediate the relationship between symptom burden and quality of life [50,51]. Because coping is potentially modifiable, routine assessment of coping strategies and individualized education to strengthen adaptive coping may be clinically useful. Cancer stigma was not independently significant in the final model after distress and coping were included, suggesting that its association with quality of life may overlap with other psychosocial processes.

Current activity level was also independently associated with quality of life. Recent studies have similarly shown that higher levels of physical activity, better physical fitness, and greater functional capacity are associated with better quality of life among patients with lung cancer undergoing chemotherapy [52,53]. Therefore, the causes of activity limitations and changes in physical functioning should be assessed from the time of diagnosis, and individualized rehabilitation and exercise programs should be implemented according to disease stage, treatment status, functional capacity, and patient tolerance.

Higher distress was associated with poorer quality of life. Recent multicenter cross-sectional and prospective studies have indicated that psychological distress is common among patients with lung cancer, may worsen during chemotherapy, and is associated with poorer quality of life and treatment outcomes [36,54]. Accordingly, regular distress screening and assessment of its physical, psychological, social, and practical causes are needed to identify high-risk patients early and provide individualized psychosocial and symptom-management interventions.

Taken together, the findings suggest that quality of life among patients with lung cancer is related to multiple domains rather than to a single factor. Sociodemographic and clinical characteristics showed associations with quality of life in the univariable analyses, while cancer stigma, distress, and cancer coping were also closely correlated with quality of life. However, the pattern of associations changed after these factors were considered simultaneously, highlighting the importance of functional status, emotional distress, and coping resources in understanding quality of life. This integrated pattern suggests that assessment and supportive care for patients with lung cancer should address both functional and psychosocial dimensions.

This study also has several strengths. First, cancer stigma, distress, cancer coping, and quality of life were examined simultaneously, allowing their interrelationships to be evaluated within a single analytic framework. Second, the hierarchical regression approach enabled assessment of the incremental contribution of each psychosocial factor beyond sociodemographic and clinical characteristics. Third, the study included both inpatients and outpatients and participants across different stages of lung cancer, thereby capturing variation in clinical and functional status. Finally, the use of established measures specific to cancer-related psychosocial experiences and quality of life enhances the clinical interpretability of the findings.

This study has several limitations. Its cross-sectional design precludes causal inference, and recruitment from a single university hospital limits generalizability. The voluntary convenience sampling approach may have introduced selection bias, and self-reported data may have introduced recall and social desirability bias. In addition, because the numbers of potentially eligible patients exposed to the recruitment notices and those who declined or did not respond were not systematically recorded, a response rate could not be calculated. This limits the ability to assess potential nonresponse bias and further constrains the generalizability of the findings. Although the total FACT-L score demonstrated acceptable internal consistency in the present study (Cronbach’s α = 0.75), the Korean validation study reported lower reliability for some subscales (as low as α = 0.52); therefore, subscale-level findings should be interpreted with caution. In addition, severely ill patients who could not complete the questionnaire were excluded, and some subgroups were small. Notably, only three participants were confined to bed throughout the day, which may have reduced the stability of estimates for that category. Residual confounding from unmeasured clinical and psychosocial factors cannot be ruled out.

The findings may be most applicable to clinically stable adults with lung cancer receiving inpatient or outpatient care in university hospital settings similar to the study site. Caution is needed when generalizing the results to patients in other healthcare settings or regions, particularly those with severe illness. Although the inclusion of patients across care settings and disease stages broadened the sample, replication in more diverse populations is needed.

## 5. Conclusions

This study examined cancer stigma, distress, cancer coping, and quality of life among patients with lung cancer. Cancer coping, current activity level, and distress were independently associated with quality of life, and the final hierarchical regression model explained 75.2% of the variance. Cancer coping showed the strongest standardized association with quality of life after adjustment for sociodemographic and clinical characteristics, cancer stigma, and distress.

These findings suggest that assessing coping, functional activity, and distress may help identify patients with lung cancer who are at risk of poorer quality of life.

Psychosocial nursing care may be strengthened by combining distress screening, support for adaptive coping, and interventions tailored to functional status while maintaining stigma-sensitive communication and family or social support. Longitudinal and intervention studies are needed to determine whether changes in these factors lead to subsequent changes in quality of life.

## 6. Implications for Nursing Practice, Education, and Research

For nursing practice, the incremental contribution of cancer coping to the final regression model highlights coping as a potentially important and modifiable target for nursing assessment and intervention. Activity level, psychological distress, and cancer coping should be considered together when assessing patients at risk of poorer quality of life. Nursing care may include regular distress screening, assessment of functional activity, support for adaptive coping, and stigma-sensitive communication that reduces self-blame and strengthens family and social support. Interventions should be individualized according to disease stage, treatment status, functional capacity, and patient preference. For nursing education, educational programs should strengthen nurses’ competencies in psychosocial assessment, distress screening, functional assessment, and communication with patients with lung cancer. Particular attention should be given to recognizing cancer-related stigma and supporting adaptive coping so that nurses can integrate physical and psychosocial needs into individualized supportive care. For research, longitudinal and intervention studies are needed to examine how changes in cancer coping, distress, and functional activity are associated with subsequent changes in quality of life and to investigate potential mediating or moderating roles of coping. Replication across multiple institutions and geographic regions is also needed to improve the generalizability of the findings.

## Figures and Tables

**Table 1:** General and Disease-related Characteristics of the Participants (N = 168).

Characteristics	Categories	*n* (%)	Mean ± SD
Gender	Male	121 (72.0)	
	Female	47 (28.0)	
Age (years)	<60	32 (19.0)	66.71 ± 8.94
	60–69	65 (38.7)	
	≥70 years	71 (42.3)	
Education level	Elementary school or less	34 (20.2)	
	Middle school	42 (25.0)	
	High school	68 (40.5)	
	College or higher	24 (14.3)	
Spouse	Yes	133 (79.2)	
	No	35 (20.8)	
Religion	Yes	66 (39.3)	
	No	102 (60.7)	
Employment	Yes	48 (28.6)	
	No	120 (71.4)	
Monthly household	<2 million	22 (13.1)	2.72 ± 1.38
income (KRW)	2–<4 million	121 (72.0)	
	≥4 million	25 (14.9)	
Perceived health	Good	53 (31.5)	
status	Moderate	56 (33.3)	
	Poor	59 (35.2)	
Time since lung	<12 months	79 (47.0)	
Cancer diagnosis	12–<36 months	37 (22.0)	
(months)	36–<60 months	16 (9.5)	
	≥60 months	36 (21.5)	
Type of lung cancer	Non-small cell lung cancer	129 (76.8)	
	Small cell lung cancer	39 (23.2)	
Cancer stage	Stage I	43 (25.6)	
	Stage II	30 (17.9)	
	Stage III	44 (26.2)	
	Stage IV	51 (30.3)	
Comorbidities	Yes	114 (67.9)	
	No	54 (32.1)	
Surgery	Yes	82 (48.8)	
	No	86 (51.2)	
Chemotherapy	Yes	137 (81.5)	
	No	31 (18.5)	
Radiation therapy	Yes	35 (20.8)	
	No	133 (79.2)	
Smoking status	Never smoked	51 (30.4)	
	Former smoker	104 (61.9)	
	Current smoker	13 (7.7)	
Current activity	As active as before the illness	19 (11.3)	
Level	Mild symptoms but fully ambulatory	78 (46.4)	
(ECOG PS-based)	Ambulatory for more than half of the day	34 (20.2)	
	Confined to bed for more than half of the day	34 (20.2)	
	Confined to bed throughout the day	3 (1.9)	
Family history of	Yes	18 (10.7)	
lung cancer	No	150 (89.3)	

**Table 2:** Levels of Cancer Stigma, Distress, Cancer Coping, and Quality of Life among Participants (N = 168).

Variables	Mean ± SD or *n* (%)	Possible Range
Cancer stigma	46.43 ± 8.46	31–124
Social isolation	17.02 ± 3.98	10–40
Stigma and shame	12.77 ± 2.81	11–44
Discrimination	6.43 ± 1.35	5–20
Smoking-related stigma	10.21 ± 2.94	5–20
Distress Thermometer	4.49 ± 2.53	0–10
<4	72 (42.9)	
≥4	96 (57.1)	
Problem List	11.38 ± 7.62	0–39
Practical	1.24 ± 1.37	0–6
Family	0.53 ± 0.91	0–4
Emotional	2.61 ± 2.04	0–6
Spiritual or religious	0.01 ± 0.07	0–1
Physical	6.98 ± 4.57	0–22
Cancer coping	56.43 ± 15.07	23–92
Intrapersonal	33.76 ± 8.62	14–56
Interpersonal	22.68 ± 7.69	9–36
Quality of life	74.02 ± 14.93	0–136
Physical	17.47 ± 7.39	0–28
Social/family	16.14 ± 5.69	0–28
Emotional	14.39 ± 5.83	0–24
Functional	13.27 ± 1.73	0–28
Lung Cancer Subscale	12.74 ± 2.75	0–28

**Table 3:** Differences in Quality of Life according to Participants’ General and Disease-Related Characteristics (N = 168).

Characteristics	Categories	Quality of Life	
		Mean ± SD	t/F (*p*)
Gender	Male	74.14 ± 14.99	0.17 (0.865)
	Female	73.70 ± 14.94	
Age (years)	<60	75.41 ± 14.03	0.44 (0.645)
	60–69	74.68 ± 14.81	
	≥70 years	72.79 ± 15.54	
Education level	Elementary school or less	68.94 ± 16.34	1.69 (0.172)
	Middle school	74.79 ± 15.44	
	High school	75.57 ± 14.08	
	College or higher	75.46 ± 13.62	
Spouse	Yes	75.25 ± 14.70	2.10 (0.037)
	No	69.34 ± 15.12	
Religion	Yes	75.33 ± 15.39	0.92 (0.360)
	No	73.17 ± 14.64	
Employment	Yes	77.58 ± 15.28	1.97 (0.050)
	No	72.59 ± 14.62	
Monthly household	<2 million	68.41 ± 16.01	2.60 (0.077)
income (KRW)	2–<4 million	74.16 ± 14.44	
	≥4 million	78.24 ± 15.44	
Perceived health	Good	85.64 ± 10.48	78.40 (<0.001)
status	Moderate	77.05 ± 10.79	
	Poor	60.70 ± 10.97	
Time since lung	<12 months	74.10 ± 15.51	0.70 (0.556)
cancer diagnosis	12–<36 months	71.27 ± 15.96	
(months)	36–<60 months	75.06 ± 12.04	
	≥60 months	76.19 ± 13.81	
Type of lung cancer	Non-small cell lung cancer	75.51 ± 14.29	2.39 (0.029)
	Small cell lung cancer	69.08 ± 16.12	
Cancer stage *	Stage I ^a^	80.05 ± 12.99	5.68 (0.001)
	Stage II ^b^	73.17 ± 14.76	d < a
	Stage III ^c^	75.61 ± 14.23	
	Stage IV ^d^	68.06 ± 15.25	
Comorbidities	Yes	72.37 ± 15.11	−2.10 (0.037)
	No	77.50 ± 14.07	
Surgery	Yes	76.16 ± 13.66	1.83 (0.070)
	No	71.98 ± 15.87	
Chemotherapy	Yes	72.04 ± 14.86	−3.74 (<0.001)
	No	82.74 ± 11.98	
Radiation therapy	Yes	73.17 ± 13.47	−0.38 (0.707)
	No	74.24 ± 15.34	
Smoking status	Never smoked	75.61 ± 15.03	0.55 (0.581)
	Former smoker	73.58 ± 15.05	
	Current smoker	71.31 ± 14.04	
Current activity	As active as before the illness ^a^	86.11 ± 10.09	30.63 (<0.001)
level *	Mild symptoms but fully ambulatory ^b^	80.73 ± 10.55	c,d,e < a,b
(ECOG PS-based)	Ambulatory for more than half of the day ^c^	68.38 ± 14.30	
	Confined to bed for more than half of the day ^d^	59.29 ± 11.15	
	Confined to bed throughout the day ^e^	53.67 ± 1.53	
Family history of	Yes	70.83 ± 13.64	−0.96 (0.340)
lung cancer	No	74.40 ± 15.08	

* Scheffé’s post hoc test. Superscript letters (a–e) denote group labels used for the post hoc comparisons shown in the table.

**Table 4:** Correlations among Cancer Stigma, Distress, Cancer Coping, and Quality of Life (N = 168).

Variables	Cancer Stigma	Distress	Cancer Coping	Quality of Life
	r			
Cancer stigma	1			
Distress	0.70 ***	1		
Cancer coping	−0.52 ***	−0.57 ***	1	
Quality of life	−0.63 ***	−0.76 ***	0.71 ***	1

*** *p* < 0.001.

**Table 5:** Hierarchical Multiple Regression Analysis of Quality of Life among Patients with Lung Cancer (N = 168).

Variables	Model 1				Model 2				Model 3				Model 4			
	B	β	*p*	95% CI	B	β	*p*	95% CI	B	β	*p*	95% CI	B	β	*p*	95% CI
(Constant)	89.29	-	<0.001	[82.548, 96.048]	110.93	-	<0.001	[100.660, 121.214]	103.48	-	<0.001	[93.279, 113.671]	74.89	-	<0.001	[62.151, 87.634]
Spouse (no) *	−1.12	−0.06	0.232	[−2.968, 0.723]	−1.04	−0.06	0.230	[−2.746, 0.666]	−1.01	−0.06	0.217	[−2.613, 0.597]	0.21	0.012	0.780	[−1.272, 1.691]
Perceived health status *																
Moderate	−5.71	−0.18	0.006	[−9.799, −1.630]	−4.23	−0.13	0.030	[−8.042, −0.411]	−3.29	−0.10	0.074	[−6.902, 0.324]	−1.51	−0.048	0.364	[−4.779, 1.763]
Poor	−16.56	−0.53	<0.001	[−21.729, −11.401]	−10.94	−0.35	<0.001	[−16.166, −5.722]	−8.04	−0.26	0.002	[−13.108, −2.962]	−4.33	−0.139	0.069	[−8.996, 0.346]
Type of lung cancer (small cell lung cancer) *	−2.83	−0.08	0.138	[−6.567, 0.916]	−1.89	−0.05	0.286	[−5.360, 1.590]	−1.41	−0.04	0.396	[−4.688, 1.865]	−1.20	−0.034	0.417	[−4.127, 1.720]
Cancer stage *																
Stage II	0.43	0.01	0.871	[−4.765, 5.623]	−0.28	−0.01	0.910	[−5.083, 4.530]	−0.78	−0.02	0.733	[−5.313, 3.744]	−1.82	−0.047	0.376	[−5.875, 2.230]
Stage III	−0.68	−0.02	0.784	[−5.591, 4.228]	0.17	0.01	0.943	[−4.382, 4.714]	−0.43	−0.01	0.844	[−4.714, 3.860]	−0.52	−0.015	0.787	[−4.348, 3.300]
Stage IV	−0.46	−0.01	0.860	[−5.551, 4.641]	0.30	0.01	0.900	[−4.418, 5.017]	−1.38	−0.04	0.547	[−5.873, 3.123]	−2.13	−0.066	0.298	[−6.145, 1.893]
Comorbidities (no) *	1.09	0.03	0.548	[−2.490, 4.675]	1.03	0.03	0.541	[−2.285, 4.336]	0.58	0.02	0.715	[−2.542, 3.700]	−0.54	−0.017	0.704	[−3.346, 2.266]
Chemotherapy (no) *	3.25	0.09	0.190	[−1.624, 8.123]	2.43	0.06	0.289	[−2.085, 6.943]	1.97	0.05	0.362	[−2.285, 6.220]	1.02	0.027	0.598	[−2.287, 4.822]
Current activity level *																
Mild symptoms but fully ambulatory	−3.27	−0.11	0.248	[−8.828, 2.297]	−0.55	−0.02	0.836	[−5.790, 4.693]	−0.02	−0.001	0.994	[−4.956, 4.919]	−4.82	−0.161	0.043	[−9.467, −0.163]
Ambulatory for more than half of the day	−7.10	−0.19	0.036	[−13.730, −0.461]	−6.80	−0.18	0.030	[−12.930, −0.668]	−3.06	−0.08	0.314	[−9.054, 2.930]	−7.06	−0.191	0.012	[−12.551, −1.573]
Confined to bed for more than half of the day	−13.82	−0.37	<0.001	[−20.804, −6.837]	−10.12	−0.27	0.003	[−16.720, −3.516]	−6.72	−0.18	0.039	[−13.101, −0.331]	−10.38	−0.280	0.001	[−16.187, −4.569]
Confined to bed throughout the day	−16.84	−0.15	0.014	[−30.247, −3.425]	−17.32	−0.15	0.006	[−29.711, −4.924]	−9.68	−0.07	0.117	[−21.805, 2.439]	−14.28	−0.127	0.011	[−25.188, −3.373]
Cancer stigma					−0.57	−0.33	<0.001	[−0.791, −0.357]	−0.26	−0.15	0.042	[−0.502, −0.009]	−0.11	−0.064	0.317	[−0.338, 0.110]
Distress									−2.16	−0.37	<0.001	[−3.100, −1.226]	−1.61	−0.272	<0.001	[−2.460, −0.752]
Cancer coping													0.37	0.370	<0.001	[0.252, 0.481]
F (*p*)	16.32 (<0.001)				19.70 (<0.001)				22.16 (<0.001)				28.61 (<0.001)			
R^2^	0.579				0.643				0.686				0.752			
Adjusted R^2^	0.544				0.611				0.655				0.726			
ΔR^2^					0.064				0.043				0.066			

* Reference groups: spouse (Yes), perceived health status (Good), type of lung cancer (non-small cell lung cancer), cancer stage (Stage I), comorbidities (Yes), chemotherapy (Yes), and current activity level (as active as before the illness). CI, confidence interval.

## Data Availability

The data presented in this study are available from the corresponding author upon reasonable request. The data are not publicly available due to privacy and ethical considerations related to the study participants.
